# The complete mitochondrial genome of rockfish *Sebastes oculatus* Valenciennes, 1833 from southwest Atlantic ocean

**DOI:** 10.1080/23802359.2019.1674704

**Published:** 2019-10-09

**Authors:** Hana Kim, Moonguen Yoon, Hyung June Kim

**Affiliations:** a Department of Taxonomy and Systematics, National Marine Biodiversity Institute of Korea, Jangsan-ro, Republic of Korea;; b Department of Biological Sciences, Inha University, Incheon, Republic of Korea

**Keywords:** Mitochondrial genome, *Sebastes oculatus*, Sebastidae, rockfish

## Abstract

The mitogenome of rockfish, *Sebastes oculatus*, has been determined for the first time. Assembled mitogenome was 16,767 bp in length, including 13 protein-coding genes, 22 transfer RNA and two ribosomal RNA genes as well as the non-coding region. The order and structure are the same as those of other *Sebastes* species. *S. oculatus* was sister to *S. nigrocinctus* and this clade is closely related with *S. rubrivinctus*, as well as support for previously published complete mitochondrial genome trees (Sandel et al. 2018). The mitogenome of *S. oculatus* provides significant DNA molecular data for further identification and phylogenetic analysis within Scorpaenid.

## Main text

The genus *Sebastes* Cuvier, 1829 called rockfishes are one of the most diverse genera of marine fishes, and their genus contains more than 110 species (Kendall 2000; Nelson 2006). Most of the *Sebastes* species are distributed in the Northern Pacific Ocean, and of these species, *S. oculatus* Valenciennes, 1833 mostly occurred off the Pacific coast of Peru and Chile to the Falkland Islands in the Atlantic Ocean (Stransky and MacLellan 2005; Nelson 2006). We report the complete mitochondrial genome (mitogenome) of *S. oculatus* for the first time, and it will be valuable information for further study on molecular taxonomy and phylogeny of the *Sebastes* species.

Specimens of *S. oculatus* were collected from the Southwest Atlantic Ocean (45°16′02.0″S 60°28′05.0″W). The voucher specimens are deposited in National Marine Biodiversity Institute of Korea (MABIK Lot no. 0009366-0009368). The genomic DNA was extracted from muscle tissue and mitogenome sequences were analysed in two ways: First, after COI gene was amplified through universal primers, primer sets designed from partial sequences of COI gene of *S. oculatus* and highly preservative gene regions for the *Sebasetes* species. Then, we conducted long-range PCR (LR-PCR) to amplify targeted genomic intervals and sequenced by the Sanger method. The sequences were assembled and annotated in comparison with previously reported mitogenome sequences of the *Sebastes* species (Jang et al. 2015; Fang et al. 2016) using Geneious v9.1.2 (Kearse et al. 2012). Additionally, we used the online Mitochondrial Genome Database of Fish server (Iwasaki et al. 2013) and tRNAscan-SE server (Lowe and Chan 2016) for annotation. Neighbor-Joining (NJ) tree was constructed to investigate the molecular taxonomic position of these species using Kimura 2-parameter model in MEGA6 (Tamura et al. 2013) and dataset used nucleotide sequences of 12 protein-coding genes (PCGs) except nad6 gene from the mitogenomes of the other 20 species in the family Sebastidae.

The circular mitogenome of *S. oculatus* (GeneBank accession number MN218776) was 16,767 bp in length, which includes two ribosomal RNA (rRNA), 22 transfer RNA (tRNA), and 13 PCGs as well as the non-coding region. Most of the genes are encoded on the H strand, while nine genes (tRAN^Gln^, tRNA^Ala^, tRAN^Asn^, tRAN^Cys^, tRAN^Tyr^, tRNA_AGN_^Serr^, nad6, tRAN^Glu^, tRAN^Pro^) are encoded on the R-strand and their order and structure in the genome are identical to those of other *Sebastes* species (Kim and Lee 2004). Twelve of 13 PCGs get off by the typical ATG as start codon, and the COI gene has GTG. Eight (nad2, COI, atp8, atp6, CO3, nad4l, nad5, nad6) of 13 PCGs use TAA for the stop codon, and two genes (nad1, nad3) ends with TAG while CO2, nad4 and cytb genes have an incomplete stop codon, T.

**Figure 1.**
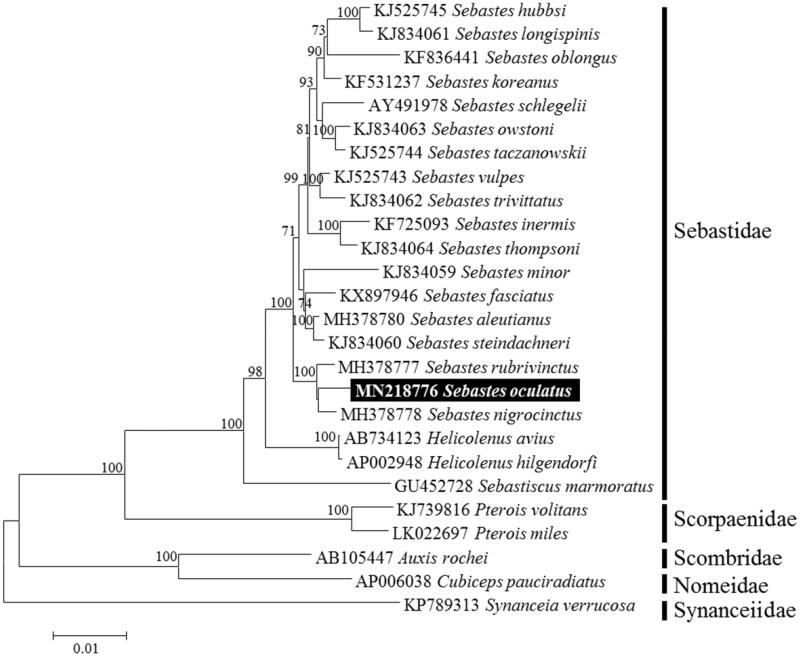
Neighbor-Joining (NJ) tree based on the 12 protein-coding genes (PCGs) except nad6 gene for 20 species of family Sabastidae including *Sebastes oculatus* and other related species under order Scorpaeniformes. *Synanceia verrucosa* (KP789313) was used as outgroup for tree rooting. Numbers above the branches indicate NJ bootstrap values from 1000 replications.

In NJ tree, *S. oculatus* was clustered with the *Sebastes* species previously announced from the GenBank, with high bootstrap values of 100% (Figure 1). *S. oculatus* was sister to *S. nigrocinctus* and this clade is closely related with *S. rubrivinctus*, as well as support for previously published complete mitochondrial genome trees (Sandel et al. 2018).
